# Establishing Medical Extended Reality Labs Within Healthcare Institutions: A Nationwide Perspective

**DOI:** 10.1177/29941520251377074

**Published:** 2025-09-12

**Authors:** Samir Ghandour, Lydia Turner, Blaire Rikard, Michaela Sawada, Suely Fazio Ferraciolli, Andre Lupp Mota, Eleni Balasalle, Raul N. Uppot, Min Lang

**Affiliations:** ^1^ Mass General Brigham Medical Extended Reality Lab, Mass General Brigham, Boston, MA, USA.; ^2^ Department of Radiology, Massachusetts General Hospital, Boston, MA, USA.; ^3^ Department of Radiology, Brigham and Women’s Hospital, Boston, MA, USA.

**Keywords:** medical extended reality, North America, health care innovation, perspective, focused interview

## Abstract

The use of extended reality (XR) technologies, including virtual reality, augmented reality, and mixed reality, has the power to transform the health care field and revolutionize medical education through its wide-ranging applications. Though the potential benefits of XR technologies are clear, the establishment of medical extended reality (MXR) Labs remains multifaceted and challenging. This work sought to better understand the process by which MXR Labs are established, the challenges faced, and ultimately the integration of the MXR technology into clinical practice. A qualitative approach was taken as focused interviews were conducted with directors from established XR research labs and centers. Thematic analysis of the interviewee responses examined the motivations for establishing an MXR Lab, the potential for MXR in health care, and the relationships between MXR Labs and industry. The resistance to MXR technology adoption and the challenge of funding during MXR Lab establishment were also explored. By providing insights into the process of establishing MXR Labs, this work seeks to help future stakeholders navigate potential obstacles more effectively, leading to a smoother path for the integration of MXR technology into medical education and clinical practice.

## Background

Extended reality (XR) technologies, encompassing virtual reality (VR), augmented reality, and mixed reality, have shown tremendous potential in transforming medical education and health care delivery. By enabling immersive, interactive experiences, XR technologies can improve a diverse range of applications in health care, including medical training, surgical planning, telemedicine, mental health, patient education, and rehabilitation.^1,2^ For instance, XR has been used to treat mental illnesses by creating immersive scenarios that can help patients face upsetting situations in a safe and controlled environment to lessen the stress when it comes to real life.^3,4^ It has also been employed in educational settings to enhance the learning experience for medical students, allowing them to practice medical procedures in a risk-free environment^5,6^; in surgical planning to enable doctors to map out their procedures in 3D images, resulting in more precise and effective surgeries^1,7^; and in developing diagnostic and treatment plans via enhanced visualization of patient disease processes.^8^

The field of medical extended reality (MXR) is still nascent and requires health care champions to demonstrate its effectiveness and to help safely integrate the technology into clinical practice. There is an increasing number of hospital-based MXR groups serving as dedicated hubs for interdisciplinary collaboration between clinicians, researchers, and technologists to enable the development, testing, and refinement of MXR tools. It provides a controlled setting for pilot studies, ensuring evidence-based integration while adhering to safety standards. However, while the need for such infrastructure and groups is obvious, the process of establishing a successful MXR group or lab within health care institutions faces many challenges.^9^ Here, we explore the importance of centralized MXR research core or lab embedded within health care systems, identify the difficulties in establishing these groups, and propose approaches to overcome these obstacles. This article presents our findings and insights from focused interviews.

## Methodology

To explore the importance of a centralized MXR research core and identify the challenges in establishing such labs, we conducted focused interviews with directors and leaders from established XR research labs and centers. A list of MXR research labs and centers in North America was compiled by the authors through Google Search. The sites were then confirmed to be affiliated with academic, tertiary, health care institutions and actively performing research in the field of MXR. Twelve directors of labs or centers were ultimately invited for an interview—six (50%) agreed to meet for a focused interview. The interviewed labs or centers’ directors were geographically located in the states of Connecticut, California, and Massachusetts.

We employed a qualitative approach using open-ended questions to gather comprehensive insights. The questions focused on several key areas: challenges and solutions, funding and support, technology and infrastructure, industry and private partnership/collaboration, evaluation and feedback, challenges in adoption and cultural shift, integration into health care delivery and education, and satisfaction with performance. Table 1 includes the key areas and themes of interest and their corresponding open-ended questions. The conclusions were derived by synthesizing recurring themes, strategies, and insights across the responses provided by the interviewees using a large language model (LLM), ChatGPT prompt tailored for this analysis. The filtered interview transcripts were annotated and fed into the LLM prompt—one category at a time—to generate an output of key recurring themes and insights. Thereafter, the authors of this piece reviewed and verified the synthesized output for accuracy and relevance in comparison with the interview transcripts and then filtered and distilled the information accordingly.

**Table 1.** Key Areas and Themes Along with Their Corresponding Question(s)

Themes	Question(s)
General	What were the primary objectives and motivations behind setting up your lab?
	How do you envision the role of MXR technologies in transforming health care?
Challenges and solutions	What were the most significant challenges you encountered while setting up your lab?
	How did you overcome these challenges? What solutions did you employ?
	Can you discuss any regulatory or compliance issues and how you addressed them?
Funding and support	How did you secure funding and institutional support for your lab?
	What strategies did you employ to gain buy-in from stakeholders and decision-makers within your institution?
Technology and infrastructure	What are the extended reality software and hardware tools that you most commonly use in your lab?
Industry and private partnership/collaboration	Do you engage with industry?
	What are the main goals of working with industry?
	What are some challenges of working with industry?
Integration and usage	How are the innovations of your lab integrated into clinical practice or patient care protocols at your institution?
	Can you share a successful case study or project demonstrating the impact of MXR technology employed by your lab?
	What are your thoughts on the regulations of the clinical applications of MXR technology and future need for FDA approval?
	What are your thoughts on the need for insurance recognition and reimbursement for MXR technology in health care?
Evaluation and feedback	How do you select your target population and their needs for the MXR technology your lab is planning on developing?
	What methods do you use to evaluate the effectiveness and impact of your innovations on students, health care professionals, or patients?
Challenges in adoption and cultural shift	Have you faced resistance or skepticism toward adopting MXR technologies among staff or students? How do you address them?

MXR, medical extended reality.

### General

Thematic analysis of the interviewee responses (*n* = 6) regarding objectives and motivations for establishing an MXR Lab revealed a shared vision of accelerating advancements in medicine by using cutting-edge technology to address gaps in clinical practice, research, education, and training. Multiple interviewees noted that their motivations stemmed from experiencing frustration with the limitations of traditional methodologies (e.g., inadequate cognitive rehabilitation tools and reliance on 2D imaging) and saw MXR technology as a solution to create more precise, immersive, and engaging patient-facing instruments that can be tailored to patient needs. Many of these labs were established from the convergence of interviewees’ personal and professional passions and evolved into hubs for innovation with the anchoring objective of generating meaningful research that results in adaptable interventions, advanced health care practices, and improved patient outcomes.

Interviewees envision MXR’s role in health care as a complementary tool to supplement existing clinical and educational frameworks for therapeutic (e.g., treating pain, anxiety, depression, ADHD, and PTSD) and instructional (e.g., elevated medical imaging and biometric acquisition, concurrent data collection, and skill development) purposes. They maintain MXR integration would not only improve the quality of telemedicine but also the accessibility and standards of health care delivery. Immersive simulations would enhance patient understanding and engagement, refine clinical training (e.g., skill practice, surgical precision, medical imaging), and support mental health therapies (e.g., modifying mental states in a reproducible manner and reducing reliance on anesthesia and pharmacological interventions). With its ability to measure multiple factors and have systematic control of stimulus delivery in realistic simulations, researchers and clinicians would be enabled to translate predictive data and contextualize interventions in real-world applications with greater impact. Interviewees anticipate the applications of MXR technology will broaden as the intersectionality of AI-driven technology and medicine evolves.

### Challenges and solutions

Funding was reportedly the most critical barrier in establishing MXR Labs, as all responses cited that substantial initial investments were needed to acquire appropriate equipment and develop software. While the initial capital cost of MXR hardware and software technology is relatively low compared with other medical devices, labs experienced difficulty appropriating such funds to effectively “kick-start” MXR research operations at a high-quality, sustainable pace. Since MXR technology is rapidly evolving and almost nonexistent at budding labs’ institutions, major equipment acquisitions need to take place in a short period of time to facilitate a lab’s establishment and operability. Furthermore, logistical issues such as securing suitable space and overcoming hardware constraints were frequently mentioned. The novelty of XR technology and its misconception as a “gamer’s tool” hindered its adoption in clinical institutions, requiring ongoing education and stakeholder engagement to address and overcome these biases. While less universally problematic, some interviewees mentioned that most regulatory challenges stemmed from difficulties in obtaining IRB approval and FDA clearance, securing CMS/insurance reimbursement, ensuring data protection in compliance with HIPPA, and addressing other considerable risks for specific groups (e.g., national security for veterans). The difficulty with the IRB process was mostly related to lack of IRB committee’s familiarity with the technology, hardware, data security, and sanitation protocols. There were some further concerns regarding the commercial companies that produce some of the headsets that have been known to collect user data. The work lay with the MXR Labs to educate the IRB committee and several groups noted that subsequent IRB submissions were more easily approved than the first.

Solutions to these challenges highlighted the importance of collaboration, adaptation, education, and resilience. Several labs relied on diversified funding through industry partnerships, combined government grants, philanthropic donations, and community involvement. Adaptive strategies, such as demonstrations showcasing and informing stakeholders and interdisciplinary teams of XR’s clinical value, were essential to gaining institutional support. Some interviewees suggested directly engaging with regulatory, compliance, and health care information groups to streamline approval processes through ongoing communication, advocating for the standardization of general guidelines related to XR terminology, hardware optimization, and safety protocols. Unfortunately, establishing industry hardware or software guidelines is more challenging as MXR is still a relatively small proportion of the overall XR audience and medical use cases of XR technology are relatively low on industry groups’ list of priorities. Ultimately, a blend of inventive problem solving, strategic networking, and community collaboration enabled labs to effectively navigate these challenges.

### Funding and support

Most interviewees shared that they secured funding and institutional support for their labs through diverse and strategic approaches, including leveraging external funding, forming interdisciplinary collaborations, and demonstrating tangible use cases to stakeholders. Moreover, they emphasized the role of government grants (e.g., NIH, CDC) and industry partnerships as primary sources of funding. Some shared that they benefited from philanthropic donations, which provided the initial resources needed for pilot studies and research infrastructure development, enabling those labs to pursue larger and more sustaining grants. Demonstrating the value of XR technology, particularly through immersive, experiential in-person demonstrations, was pivotal in gaining buy-in from institutional leadership and decision-makers. This allowed MXR leaders to address emerging skepticism in MXR technology and its potential, particularly by showcasing its potential in addressing real-world challenges such as pain management and public health education.

The respondents expressed several strategies to gain stakeholder buy-in. The most notable strategy was highlighting the importance of personal engagement and creating a visible impact among end-users. Specific examples include inviting decision-makers to experience XR applications firsthand and aligning the technology with institutional priorities, such as addressing the opioid crisis or improving educational tools. Some even capitalized on grassroots efforts, such as lending out headsets and conducting workshops to generate interest within their immediate catchment areas. Documenting successes, sharing patient stories, and emphasizing the innovative potential of XR were important techniques employed to further solidify institutional support. The combination of targeted outreach, strategic demonstrations, and alignment with institutional and societal goals created a strong foundation for sustained funding and stakeholder engagement.

### Technology and infrastructure

The interviewees expressed that their labs utilize a wide array of XR hardware and software tools, often selecting devices and programs based on their versatility, availability, price, and relevance to specific projects. Many reported to being “hardware-agnostic,” experimenting with a variety of headsets including the Meta Quest (2 and 3), Pico G2, HoloLens, and Apple Vision Pro. These devices cater to different needs, from cost-effectiveness to advanced features such as heart rate and pupillometry tracking. Labs also incorporate motion capture systems such as OptiTrack for prototyping and spatial audio for immersive experiences. Most importantly, the interviewees emphasized that the selection of hardware is typically project-driven, which ensures that the devices align with the intended application, whether clinical or educational.

On the software side, Unity emerged as the dominant development platform utilized by the interviewees and their labs due to its cross-platform compatibility and widespread adoption for immersive environment development for research applications, clinical use, or education. Some labs developed their own homegrown software to meet specific research and clinical needs, allowing them to create tools such as cognitive therapy apps and mental health support programs. Additionally, FDA-approved technology, such as Applied VR’s RelieVRx, was noted for its integration into therapeutic clinical trials for chronic pain. The accessibility of such development tools and the rapid evolution of XR technology have made these innovations increasingly feasible, enabling labs to explore novel applications while adapting to the unique requirements of their institutions and target populations.

### Industry and private partnership/collaboration

Collaborations between XR labs and industry partners are instrumental in driving innovation by providing essential funding, access to advanced technologies, and resources for large-scale trials. The interviewees generally expressed that these partnerships facilitate the transition of XR tools from research-grade setups to practical, patient-ready solutions. By working closely with industry, their labs were able to align their projects with user needs, leveraging the latest tools such as the Apple Vision Pro to improve patient outcomes and expand applications beyond traditional boundaries. This collaborative approach also fosters the development of user-friendly and commercially viable software that enhances adoption and usability.

However, these partnerships come with challenges, such as aligning academic and corporate goals. Labs may face financial constraints, intellectual property restrictions, and conflicts between research rigor and industry priorities. To overcome these barriers, the respondents attempted to foster “win-win relationships” with a shared emphasis on patient benefits. Success in these collaborations requires balancing the academic pursuit of evidence-based innovation with the commercial drive for rapid development, ensuring that breakthroughs remain both ethical and impactful.

### Integration and usage

The integration of MXR technologies into clinical practice showcases remarkable versatility, with predominant applications in pain management, mental health, and surgical guidance. By embedding these tools into existing workflows, MXR Labs aim to minimize disruption and maximize clinical utility. Practical examples shared by the interviewees include VR for cancer infusion centers, pediatric care, labor and delivery, and surgical precision in breast surgery. These initiatives highlight MXR’s potential to enhance patient experiences, improve clinical outcomes, and complement traditional medical treatments.

Despite promising applications, implementation remains challenging due to compliance issues, logistical and information technology (IT) barriers, and the need for education to encourage adoption. A lack of standardized method of IT integration, data collection, and storage, wireless network security has made it challenging for some groups to deploy MXR tools in the clinical setting. Some have opted for wired hardware to avoid network security issues and storage of all data on a secure cloud platform and not on the hardware directly. Stakeholder engagement plays a vital role in addressing these barriers, emphasizing simplicity and practical relevance in MXR tools. Moreover, financial concerns such as the need for insurance reimbursement and regulatory clarity, like FDA approval, need to be better addressed before widespread integration. Demonstrating cost-effectiveness and tangible return on investment and benefits will be essential to solidify MXR’s position in health care.

### Evaluation and feedback

Interviewees employ user-centered design and iterative feedback processes to ensure their innovations address unmet needs and provide benefits beyond traditional means. Co-development with target populations, informed by input from stakeholders such as patients, educators, and medical staff, enabled the respondents to create tailored solutions. For instance, an interviewee shared that user feedback prompted her to transition from handwashing education to germ-spread awareness in the NICU settings as the main focus of her laboratory’s application development.^10^

To evaluate effectiveness, laboratories combined qualitative and quantitative methods, such as feasibility studies, hybrid trials, and randomized controlled trials. Continuous feedback from advisory boards and specific user groups ensures that the solutions remain relevant and scalable. Balancing rigorous scientific evaluation with practical considerations, such as cost and accessibility, allows laboratories to deliver impactful tools that address both clinical and community needs. This iterative process bridges the gap between experimental success and real-world application.

### Challenges in adoption and cultural shift

Adopting MXR technologies faces significant resistance, particularly among older practitioners and administrators who are skeptical of their clinical utility and cost-effectiveness. The association of these tools with gaming often creates doubts about their seriousness in medical applications. Respondents used strategies such as hands-on demonstrations and presenting patient success stories to address these concerns, fostering curiosity and gradual acceptance among hesitant members of the medical community. Additionally, focusing on ROI and usability further helps mitigate resistance from administrators and practitioners.

Generational differences also play a key role in adoption dynamics. Younger staff and students readily embrace MXR tools, often bringing innovative ideas and energy to their implementation. On the contrary, older practitioners require tailored strategies, including simplified interfaces and user-friendly designs, to reduce adoption barriers. By combining education, exposure, and cultural adaptation, MXR technologies can be integrated more seamlessly across diverse audiences, ultimately transforming health care practices.

## Summary

Our focused interviews with leaders of established MXR Labs helped uncover the motivations behind creating these innovative hubs, the challenges faced during their establishment, and the strategies employed to overcome them. Table 2 provides insight summaries we gleaned from conducting these focused interviews for each category. MXR technologies offer immense promise, from advancing mental health therapies and surgical planning to enhancing patient education and medical training. However, successful integration into academic health care institutions requires overcoming barriers, such as funding constraints, skepticism, and regulatory hurdles. By leveraging interdisciplinary collaborations, fostering industry partnerships, and employing evidence-based practices, MXR Labs may bridge the current gap between research and real-world application. Moreover, the interviewees emphasize the importance of user-centered design, cultural adaptation, and iterative feedback in ensuring the effectiveness and scalability of MXR tools under development, ultimately advocating for their role as indispensable instruments in the evolution of modern health care.

**Table 2.** Key Insight Summaries from Each Major Category Highlighted in the Focused Interviews

Category	Insights
General	MXR Labs were established primarily to address gaps in clinical and educational practices, leveraging XR technology for innovative, patient-centered solutions. Such labs were envisioned as transformative hubs to integrate immersive learning, telemedicine, and AI to enhance health care delivery and improve patient outcomes.
Challenges and solutions	The key challenges in establishing MXR Labs included securing funding, managing regulatory oversight, and bridging the gap between the slow pace of academia and the fast-moving tech industry. Solutions involved creating sustainable funding strategies, proactive communication with regulatory bodies, and fostering interdisciplinary collaborations to overcome institutional and logistical barriers.
Funding and support	Securing funding relied on diverse sources such as federal grants, philanthropic donors, and industry collaborations, often driven by demonstrating the relevance and potential of MXR technology via pilot testing. Stakeholder buy-in was achieved by showcasing real-world use cases, leveraging institutional priorities, and emphasizing the strategic alignment of MXR with health care innovation goals.
Technology and infrastructure	MXR Labs use a variety of hardware (e.g., Meta Quest, Apple Vision Pro) and software (e.g., Unity, custom-built, in-house applications) tailored to specific clinical and educational needs. The focus is on integrating user-friendly, adaptable tools with interdisciplinary collaboration to ensure effective deployment in diverse health care scenarios.
Industry and private partnership/collaboration	Industry partnerships enable MXR Labs to secure funding, gain early access to emerging technologies, and expedite commercialization. However, challenges such as conflicting goals, intellectual property disputes, and varying operational tempos necessitate careful negotiation to ensure mutually beneficial outcomes for both parties involved.
Integration and usage	Integrating MXR into clinical practice can be tricky, involving pilot programs, IRB-approved studies, and quality improvement projects across multiple specialties. Successful cases highlight improved patient engagement, reduced pain, or surgical precision, though challenges persist in aligning regulatory standards and achieving insurance coverage.
Evaluation and feedback	MXR technology is developed through a user-centered design process, identifying unmet needs and tailoring solutions in collaboration with stakeholders. Effectiveness is evaluated through metrics such as usability, engagement, and clinical outcomes, ensuring iterative refinement and real-world applicability.
Challenges in adoption and cultural shift	Resistance to MXR technology adoption, particularly among older practitioners and administrators, is often rooted in skepticism about its utility and ROI. Overcoming this requires hands-on demonstrations, evidence-based results, and user-friendly designs, with younger generations showing greater enthusiasm for embracing the technology.

AI, artificial intelligence; MXR, medical extended reality; XR, extended reality.

## Abbreviations Used

ADHD: Attention deficit hyperactivity disorder
AR: Augmented reality
CDC: Centers for Disease Control and Prevention
CMS: Centers for Medicare & Medicaid Services
FDA: Food and Drug Administration
HIPAA: Health Insurance Portability and Accountability Act
IRB: Institutional review board
IT: Information Technology
LLM: Large language model
MXR: Medical extended reality
NICU: Neonatal Intensive Care Unit
NIH: National Institutes of Health
PTSD: Post-traumatic stress disorder
ROI: Return on investment
VR: Virtual reality
XR: Extended reality
